# Outcomes and Predictors of In-Hospital Mortality After Isolated Coronary Artery Bypass Grafting in Patients with Severe Ischemic Cardiomyopathy: A Single-Centre Retrospective Analysis

**DOI:** 10.3390/medsci14020216

**Published:** 2026-04-27

**Authors:** Mustafa Al-Obaidi, Andreas Winter, Afsaneh Karimian-Tabrizi, Thomas Walther, Fabian Emrich

**Affiliations:** Department of Cardiovascular Surgery, University Hospital, Goethe University Frankfurt, Theodor-Stern-Kai 7, 60590 Frankfurt am Main, Germany

**Keywords:** coronary artery bypass, myocardial revascularization, ischemic cardiomyopathy, ventricular dysfunction left, mortality, risk factors

## Abstract

Background: Patients with coronary artery disease (CAD) and severely reduced left ventricular ejection fraction (LVEF) ≤ 30% represent a high-risk group for coronary artery bypass grafting (CABG). Outcomes are of significant concern; therefore, real-world outcome data and predictors of early mortality remain important for perioperative decision-making. Aim: This study aims to evaluate early and mid-term outcomes in patients with severe ischemic cardiomyopathy (LVEF ≤ 30%) undergoing isolated CABG and to identify independent predictors of in-hospital mortality. Methods: We conducted a retrospective single-centre cohort analysis including patients with preoperative LVEF ≤ 30% undergoing isolated CABG (2017–2021). Early outcomes included in-hospital and 30-day mortality. The mid-term outcome was all-cause mortality up to 36 months. Multivariable logistic regression was used to identify independent predictors of in-hospital mortality. A predefined subgroup comparison was performed for LVEF ≤ 20% versus LVEF 20–30%. Results: The study comprised 147 patients with LVEF ≤ 30% undergoing isolated CABG. Overall, in-hospital mortality was 21.1%, and 30-day mortality was 23.8%. Cumulative all-cause mortality was 31.3%, 37.4%, and 42.9% at 12, 24 and 36 months respectively. Patients with LVEF ≤ 20% showed significantly higher early mortality (in-hospital 37.2% vs. 14.4%; 30-day 41.9% vs. 16.3%) and 36-month mortality (58.1% vs. 36.5%) compared with those with LVEF at 20–30%. Independent predictors of in-hospital mortality included preoperative hemodynamic instability, elevated operative risk scores, emergency status, prolonged cardiopulmonary bypass time, and major postoperative complications (all *p* < 0.05). Conclusions: CABG in patients with LVEF ≤ 30% is associated with substantial early mortality, while mid-term survival remains acceptable. Severely reduced LVEF identifies a particularly high-risk subgroup requiring careful perioperative risk stratification.

## 1. Introduction

Cardiovascular diseases remain the leading cause of death in industrialized countries (accounting for roughly one-third of all deaths) [1] and represent a major contributor to morbidity and mortality in Europe [2,3]. Coronary artery disease (CAD) represents the largest fraction of this burden (around 40% in the USA and 35% in Germany) [1,2] and is the underlying cause of ischemic cardiomyopathy (ICM), which is the most common cause of heart failure in Western nations [4,5]. Patients with severe left ventricular dysfunction (LVD), typically defined as LVEF ≤ 30% [6], due to CAD, represent a high-risk but potentially high-benefit group for revascularization [7].

Based on the 2018 guideline of the European Society of Cardiology (ESC) and European Association for Cardio-Thoracic Surgery (EACTS) on myocardial revascularization and in addition to optimal medical therapy (OMT), revascularization is recommended in patients with CAD and severe LVD (Class Ib recommendation), as it improves symptoms, survival, and prognosis through enhanced perfusion of hibernating myocardium compared with OMT alone [8]. However, the optimal revascularization strategy, either coronary artery bypass grafting (CABG) or percutaneous coronary intervention (PCI), is not clearly determined.

Randomized evidence evaluating revascularization strategies in patients with severe LVD remains limited [8,9], as most trials excluded this high-risk population. In a large prospective registry of patients with multivessel CAD and severe LVD, PCI using everolimus-eluting stents demonstrated survival comparable to CABG at mid-term follow-up (mean follow-up 2.9 years); however, PCI was associated with higher rates of myocardial infarction and repeat revascularization, whereas CABG carried a higher risk of stroke [10]. The overall prognostic benefit of PCI in severe LVD remains uncertain [11]. Notably, the REVIVED randomized trial showed no significant improvement in clinical outcomes with PCI plus OMT compared with OMT alone in patients with ICM [12].

In contrast, CABG has consistently demonstrated survival and symptomatic benefits over medical therapy in patients with ischemic LVD. The STICH trial [13] and its 10-year follow-up [14] showed a significant long-term survival advantage of surgical revascularization compared with optimal medical therapy alone in patients with LVEF ≤ 35%. Consequently, current guidelines strongly recommend CABG as the preferred strategy in patients with multivessel CAD and severe LV dysfunction. The 2018 ESC/EACTS guidelines assign a Class I indication for CABG in this setting [8], reaffirmed by the 2024 ESC/EACTS Guidelines for chronic coronary syndromes [15] and reflected in American Heart Association (AHA)/American College of Cardiology (ACC) recommendations [11]. In this context, these guidelines emphasize the role of the multidisciplinary Heart Team evaluation and assessment to guide revascularization strategy, particularly in patients with complex coronary anatomy and severe left ventricular dysfunction. The Heart Team integrates clinical status, anatomical complexity, myocardial viability, comorbidities, surgical risk, and patient preferences to determine the optimal approach.

Despite advances in surgical techniques and perioperative care that have reduced CABG-related mortality [16], as well as improvements in heart failure therapy [17], patients with LVEF ≤ 30%, advanced heart failure, or hemodynamic instability remain at substantially increased operative risk [18]. Their prognosis under conservative management is poor [13], and early mortality after CABG remains substantially higher than in patients with preserved ejection fraction (EF) [19,20,21]. Moreover, the proportion of patients with advanced heart failure referred for CABG continues to increase [22], underscoring the importance of refined risk stratification and patient selection.

Because such high-risk patients are often underrepresented in randomized trials (e.g., STICH [13]), real-world data are essential. Contemporary evidence on early and mid-term mortality after CABG in severe ischemic cardiomyopathy remains limited, highlighting the need for outcome data reflecting the full clinical risk spectrum.

This highlights a critical gap in the literature, particularly regarding real-world outcomes in high-risk patients with severe LVD undergoing surgical revascularization.

The present study aims to evaluate early and mid-term outcomes in patients with severe ischemic cardiomyopathy (LVEF ≤ 30%) undergoing isolated CABG at a tertiary cardiac surgery centre in Germany. Furthermore, we sought to identify independent predictors of in-hospital mortality and to compare outcomes between patients with LVEF ≤ 20% and those with LVEF 20–30%, providing clinically relevant insights into perioperative risk assessment in this high-risk population.

## 2. Materials and Methods

### 2.1. Study Design and Setting

This study was designed as a single-centre retrospective observational analysis conducted at the Department of Cardiovascular Surgery, University Hospital Frankfurt/Main, Germany. All consecutive adult patients with CAD and severely reduced left ventricular ejection fraction (LVEF ≤ 30%) who underwent isolated CABG between January 2017 and December 2021 were included. The study was performed in accordance with the Declaration of Helsinki and was approved by the local institutional ethics committee (Geschäftsnummer: 2023-1334). Due to the retrospective design, the requirement for written informed consent was waived.

### 2.2. Study Population

The study population consisted of patients diagnosed with CAD and severe LVD (LVEF ≤ 30%) who underwent isolated surgical myocardial revascularization using CABG, performed either with or without cardiopulmonary bypass. The indication for CABG was based on a multidisciplinary Heart Team assessment, integrating coronary anatomy, clinical presentation, operative risk, and current guideline recommendations.

Patients were stratified into two predefined subgroups based on preoperative left ventricular function:

- Patients with very severe dysfunction (LVEF ≤ 20%);
- Patients with severe dysfunction (LVEF 21–30%).

### 2.3. Inclusion and Exclusion Criteria

Patients were eligible for inclusion if they had angiographically confirmed coronary artery disease, a preoperative LVEF ≤ 30%, and underwent isolated CABG. Patients were excluded if they had undergone previous cardiac surgery, required concomitant valve surgery (including moderate or severe mitral regurgitation), ventricular aneurysmectomy, or any additional open-heart procedures.

All included patients had complete clinical and outcome data available; therefore, no cases were excluded due to incomplete records.

### 2.4. Preoperative Assessment

All patients underwent a standardized preoperative evaluation including clinical examination, laboratory testing, coronary angiography, and transthoracic echocardiography (TTE). Echocardiographic examinations were performed by experienced cardiologists trained in perioperative echocardiography, and left ventricular ejection fraction was assessed using standard echocardiographic techniques. Severe left ventricular dysfunction was defined as an LVEF ≤ 30%, in accordance with the 2015 recommendations of the American Society of Echocardiography and the European Association of Cardiovascular Imaging [6].

### 2.5. Data Collection

Clinical data were retrospectively obtained from institutional electronic medical records, including the ORBIS^®^ and MetaVision^®^ systems.

Collected preoperative variables included demographic characteristics, cardiovascular risk factors and comorbidities, clinical heart failure status according to the New York Heart Association (NYHA) classification, angina severity according to the Canadian Cardiovascular Society (CCS) classification, history of myocardial infarction or cardiopulmonary resuscitation (CPR), preoperative inotropic support, laboratory parameters including renal function and troponin status, and operative risk assessment using EuroSCORE II. Coronary anatomy was documented with respect to the number of diseased vessels and the presence of left main stem disease. Information on preoperative medical therapy was available in the clinical records; however, detailed and standardized data on specific medication regimens were not consistently documented and were therefore not included in the analysis.

Intra-operative variables comprised urgency of surgery, surgical technique (on-pump or off-pump CABG), number of bypass grafts, use of the internal thoracic artery, cardiopulmonary bypass and aortic cross-clamp times, and the use and type of mechanical circulatory support devices, including intra-aortic balloon pump (IABP) and extracorporeal membrane oxygenation (ECMO).

Postoperative variables included intensive care unit and hospital length of stay, major postoperative complications such as low cardiac output syndrome, acute renal failure and need for renal replacement therapy, pneumonia, cerebrovascular events, arrhythmias, severe bleeding requiring re-intervention, and sternal or vascular complications. In-hospital, 30-day and up to 36-month all-cause mortality were documented for all patients.

### 2.6. Study Endpoints

The primary endpoints of the study were in-hospital and 30-day all-cause mortality. Secondary endpoints included postoperative complications, duration of intensive care unit (ICU) and hospital stay, mid-term mortality and survival up to 36 months, and identification of independent predictors of in-hospital mortality.

### 2.7. Statistical Analysis

Statistical analyses were performed using SPSS software (Version 29, IBM Corp., Armonk, NY, USA). Continuous variables are presented as the mean ± standard deviation or median with range, as appropriate, while categorical variables are expressed as absolute numbers and percentages. Comparisons between groups were conducted using Student’s *t*-test or the Mann–Whitney *U* test for continuous variables and the chi-square test or Fisher’s exact test for categorical variables.

Survival analyses were performed using the Kaplan–Meier method, and differences between groups were assessed with the log-rank test. Univariate logistic regression analyses were initially performed to identify variables associated with in-hospital mortality. Variables with a *p*-value < 0.20 in univariate analysis or deemed clinically relevant were subsequently entered into a multivariable logistic regression model. Results are reported as odds ratios with 95% confidence intervals, and a two-sided *p*-value < 0.05 was considered statistically significant.

## 3. Results

### 3.1. Overall Overview

Between 2017 and 2021, 147 patients with CAD and severe LVD (LVEF ≤ 30%) underwent isolated CABG and were included in the analysis. Mean age was 66.6 ± 9.9 years (*n* = 147), and 84.4% (*n* = 124) were male. Three-vessel CAD was present in 93.9% (*n* = 138) and left main stem involvement was observed in 51.7% (*n* = 76). Preoperatively, 76.9% (*n* = 113) of patients were in NYHA class III–IV, 34.0% (*n* = 50) were in CCS class IV, 56.5% (*n* = 83) had a EuroSCORE II > 8% (high-risk), 59.9% (*n* = 88) had positive troponin levels, 33.3% (*n* = 49) required inotropic support, and 15.0% (*n* = 22) underwent CPR prior to surgery. Urgent or emergency procedures accounted for 62.6% (*n* = 92) of operations. On-pump CABG was performed in 79.6% of patients (*n* = 117), with left internal thoracic artery use in 92.5% (*n* = 136) and a mean of three grafts per patient (58.5%, *n* = 86).

Postoperatively, low cardiac output syndrome (LCOS) occurred in 63.3% (*n* = 93), acute renal failure occurred in 28.6% (*n* = 42), pneumonia occurred in 26.5% (*n* = 39), postoperative dialysis was required in 25.2% (*n* = 37), and 23.1% of patients (*n* = 34) developed arrhythmia. Less frequent complications were observed, including severe bleeding (12.9%, *n* = 19), wound healing problems (7.5%, *n* = 11), arterial vascular complications (6.1%, *n* = 9), sternal instability (4.8%, *n* = 7), and bypass revision (2.7%, *n* = 4). Mean ICU stay was 6.9 ± 7.7 days, and mean hospital stay was 14.5 ± 10.7 days. In-hospital mortality was 21.1% (*n* = 31), and 30-day mortality was 23.8% (*n* = 35). Cumulative mortality was 31.3% (*n* = 46) at 12 months, 37.4% (*n* = 55) at 24 months, and 42.9% (*n* = 63) at 36 months.

### 3.2. Subgroup Analysis According to Left Ventricular Ejection Fraction

#### 3.2.1. Baseline Characteristics and General Distribution

The cohort was stratified into patients with LVEF < 20% (29.3%, *n* = 43) and those with LVEF 20–30% (70.7%, *n* = 104).

**Figure 1:**
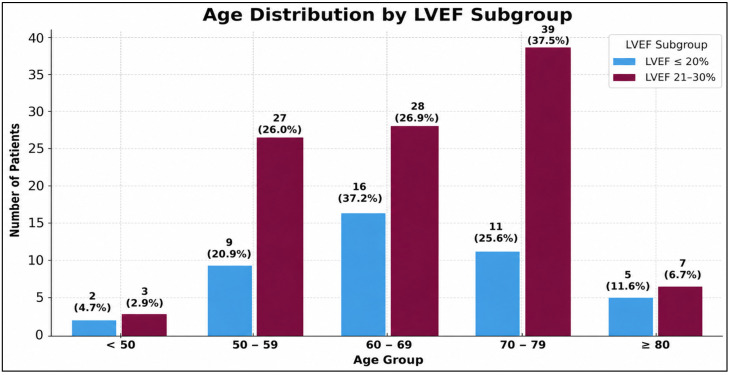
Distribution of age by LVEF subgroup.

Male predominance was observed in both groups (81.4% vs. 85.6%). Age distribution was similar between subgroups, with most patients aged 50–79 years; patients with EF < 20% were more frequently aged 60–69 years, whereas those with EF 20–30% were more commonly aged 70–79 years (Figure 1).

Baseline comorbidities were highly prevalent in both subgroups (Table 1). Diabetes mellitus (44.2% (*n* = 19) vs. 42.3% (*n* = 44)) and hypertension (53.5% (*n* = 23) vs. 61.5% (*n* = 64)) were common and similarly distributed. Lung disease, predominantly chronic obstructive pulmonary disease (COPD), occurred more frequently in patients with EF < 20% (18.6% (*n* = 8) vs. 12.5% (*n* = 13)). Preoperative renal failure was more common in the EF < 20% group (14.0% (*n* = 6) vs. 5.8% (*n* = 6)). Neurologic disease was infrequent in both groups. Mean Body Mass Index (BMI) was comparable between subgroups (26.95 ± 5.4 vs. 27.70 ± 4.4 kg/m^2^).

In addition, analysis of the cumulative burden of preoperative risk factors (diabetes mellitus, arterial hypertension, lung disease, neurologic disease, renal failure, and BMI ≥ 25 kg/m^2^) showed that only 6.1% (*n* = 9) of patients had no identifiable risk factor, whereas 93.9% (*n* = 138) presented with at least one risk factor. Multiple concurrent risk factors were common, with 69.4% (*n* = 102) of patients exhibiting two or more preoperative risk factors and almost one-third having three or more, reflecting the high-risk clinical profile of the study population (Table 2).

#### 3.2.2. Clinical Characteristics of Patients According to LVEF Subgroup

##### Preoperative Characteristics

**Figure 2:**
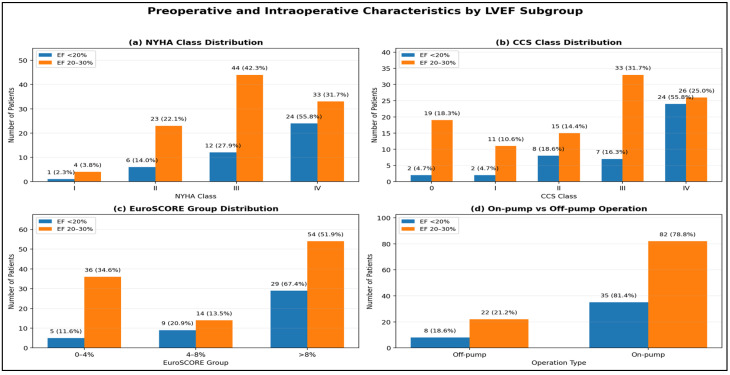
Distribution of preoperative and intra-operative characteristics by LVEF subgroup; data are presented as numbers (%). (**a**) NYHA class distribution; (**b**) CCS angina class distribution; (**c**) Euroscore II group distribution; (**d**) on-pump versus off-pump operation. LVEF = left ventricular ejection fraction; NYHA = New York Heart Association functional class; CCS = Canadian Cardiovascular Society.

Preoperative characteristics differed between LVEF subgroups (Table 3). Positive preoperative troponin was more frequent in patients with LVEF < 20% (74.4%, *n* = 32) than in those with LVEF 20–30% (53.8%, *n* = 56) and preoperative inotropic support was also more common in the LVEF < 20% group (53.5% (*n* = 23) vs. 25.0% (*n* = 26)). Patients with LVEF < 20% more frequently presented with NYHA class IV and CCS class IV symptoms (Figure 2a,b), and a higher proportion had EuroSCORE II > 8% (Figure 2c).

Regarding cardiac characteristics (Table 4), recent myocardial infarction (<2 days) occurred more often in patients with LVEF < 20% (58.1%, *n* = 25) compared to those with LVEF 20–30% (35.6%, *n* = 37). Three-vessel CAD was common in both groups (95.3% (*n* = 41) vs. 93.3% (*n* = 97)), as well as left main stem disease (55.8% (*n* = 24) vs. 50.0% (*n* = 52)). An abnormal preoperative rhythm, prior PCI, and carotid artery stenosis showed similar distributions between subgroups.

##### Intra-Operative Surgical Information and Its Association with Preoperative LVEF

Surgical Information differed according to LVEF (Table 5). Patients with LVEF < 20% more often underwent urgent surgery, including procedures after CPR (34.9% (*n* = 15) vs. 5.8% (*n* = 6); *p* = 0.001). Mechanical circulatory support was also used more often (51.2% (*n* = 22) vs. 18.3% (*n* = 19); *p* = 0.007), with longer support duration (2.33 ± 3.2 vs. 1.14 ± 3.4 days; *p* = 0.046). Aortic cross-clamp time was similar, whereas cardiopulmonary bypass time was longer in patients with LVEF < 20% (119.67 ± 72.0 vs. 87.7 ± 53.4 min; *p* = 0.011). Use of the left internal thoracic artery, number of grafts, and on-pump procedures (Figure 2d) were comparable between subgroups.

##### Postoperative Outcome According to LVEF Subgroup

Hospitalization

Postoperative hospitalization varied between LVEF subgroups (Table 6). Patients with LVEF < 20% had a longer ICU stay (8.1 ± 7.6 days) compared to those with LVEF 20–30% (6.4 ± 7.7 days; *p* = 0.033), whereas total hospital length of stay (LOS) was shorter in the LVEF < 20% group (12.2 ± 8.5 vs. 15.4 ± 11.3 days; *p* = 0.029).

Complications

Postoperative complications are summarized in Table 7. LCOS (83.7% (*n* = 36) vs. 54.8% (*n* = 57); *p* = 0.001). Acute renal failure (41.9% (*n* = 18) vs. 23.1% (*n* = 24); *p* = 0.028), postoperative renal replacement therapy (37.2% (*n* = 16) vs. 20.2% (*n* = 21); *p* = 0.038), pneumonia (39.5% (*n* = 17) vs. 21.2% (*n* = 22); *p* = 0.026), emergency re-operation (34.9% (*n* = 15) vs. 13.5% (*n* = 14); *p* = 0.005), and life-threatening postoperative bleeding (25.6% (*n* = 11) vs. 7.7% (*n* = 8); *p* = 0.006) were all significantly more common in the LVEF < 20% subgroup. Other complications, listed in Table 7, showed no significant differences between groups.

**Figure 3:**
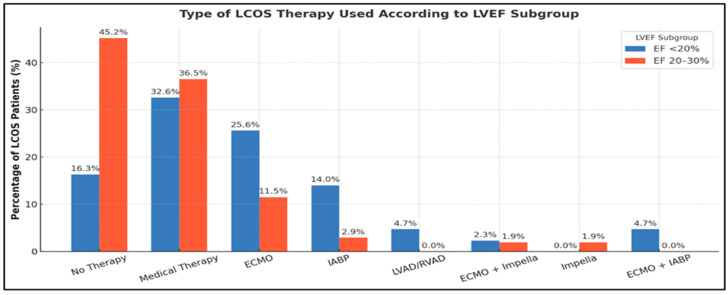
Type of therapeutic strategies for LCOS according to LVEF subgroup. Distribution of therapeutic strategies used in patients who developed postoperative LCOS, stratified by preoperative LVEF (<20% vs. 20–30%). LCOS, low cardiac output syndrome; LVEF, left ventricular ejection fraction; ECMO, extracorporeal membrane oxygenation; IABP, intra-aortic balloon pump; LVAD, left ventricular assist device; RVAD, right ventricular assist device.

Among patients who developed postoperative LCOS, advanced mechanical circulatory support was more frequently required in the LVEF < 20% subgroup, with higher use of extracorporeal membrane oxygenation (ECMO) and an intra-aortic balloon pump (IABP), whereas patients with LVEF 20–30% were more often managed with medical therapy alone and required no additional support. The distribution and intensity of LCOS therapy according to LVEF subgroup are detailed in Figure 3.

#### 3.2.3. Mortality According to LVEF Subgroup

Mortality outcomes according to the LVEF subgroup are summarized in Table 8 and illustrated using Kaplan–Meier survival curves for in-hospital, 30-day, and mid-term follow-up.

##### In-Hospital Mortality

In-hospital mortality occurred in 21.1% of the overall cohort (31/147) and was significantly higher among patients with LVEF < 20% compared to those with LVEF 20–30% (37.2% (16/43) vs. 14.4% (15/104); *p* = 0.024) (Table 8).

**Figure 4:**
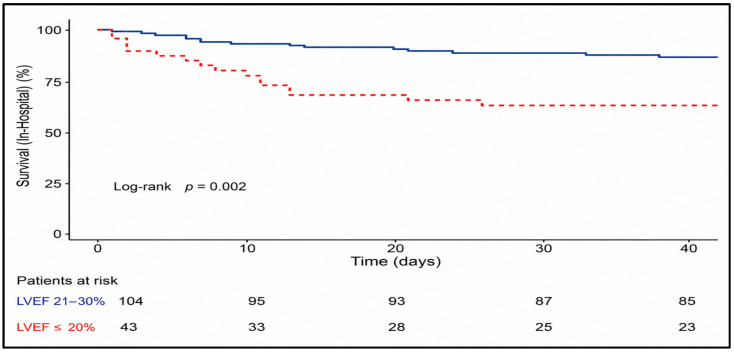
Kaplan–Meier survival curve for in-hospital mortality according to the LVEF subgroup. Survival probability during the index hospitalization is shown for patients with LVEF < 20% and LVEF 20–30%. Patients with LVEF < 20% exhibited a markedly lower in-hospital survival, with a steep early decline shortly after surgery compared to patients with LVEF 20–30%.

Kaplan–Meier analysis of in-hospital survival demonstrated a pronounced early warning in patients with LVEF < 20%, with a steep decline in survival probability shortly after surgery. In contrast, patients with LVEF 20–30% exhibited a more gradual decline in survival, maintaining a substantially higher probability of in-hospital survival throughout the hospitalization period (Figure 4).

##### Early (30-Day) Mortality

Early postoperative mortality within 30 days was observed in 23.8% of all patients (35/147) (Table 8). Mortality at 30 days remained significantly higher in patients with LVEF < 20% (41.9% (18/43) vs. 16.3% (17/104); *p* = 0.002).

**Figure 5:**
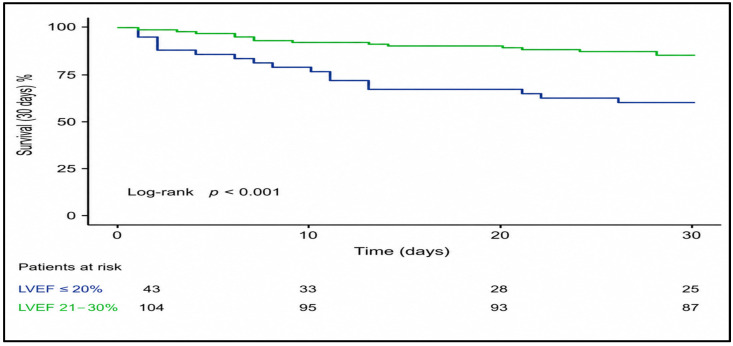
Kaplan–Meier survival curve for 30-day mortality according to the LVEF subgroup. Patients with LVEF < 20% demonstrated significantly reduced early survival, with most events occurring within the first postoperative weeks, whereas patients with LVEF 20–30% maintained a higher probability of 30-day survival.

Kaplan–Meier survival curve for the 30-day period revealed an early and sustained separation between the two LVEF subgroups, with the majority of deaths in the LVEF < 20% group occurring within the first postoperative weeks. Patients with LVEF 20–30% showed a higher early survival probability, with fewer events distributed more evenly over the 30-day follow-up (Figure 5).

##### Mid-Term Mortality at 12, 24, and 36 Months

Mid-term mortality increased progressively during follow-up and varied markedly according to preoperative LVEF (Table 8). Among patients with LVEF at 20–30%, cumulative mortality was 23.1% (24/104), 29.8% (31/104), and 36.5% (38/104) at 12, 24, and 36 months respectively. In contrast, patients with LVEF < 20% demonstrated substantially higher mortality at all time points, reaching 51.2% (22/43), 55.8% (24/43, and 58.1% (25/43) at 12, 24 and 36 months.

**Figure 6:**
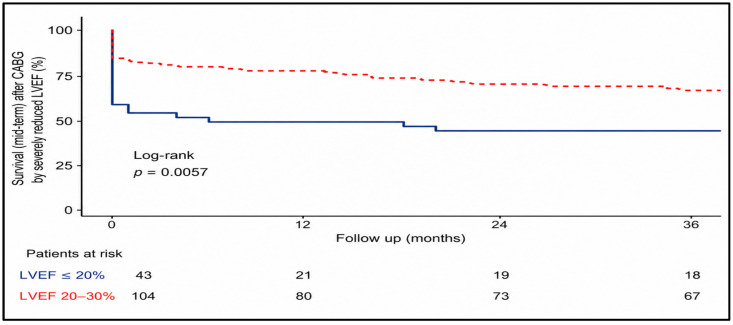
Kaplan–Meier survival curve for mid-term follow-up according to the LVEF subgroup. Mid-term survival up to 36 months after CABG is shown for patients with LVEF < 20% and LVEF 20–30%. An early separation of survival curves was observed, persisting throughout follow-up, indicating consistently lower survival in patients with severely reduced LVEF.

Kaplan–Meier survival analysis confirmed significantly reduced mid-term survival in patients with LVEF < 20%, with an early divergence of survival curves that persisted throughout the follow-up period. While mortality continued to increase over time in both subgroups, the survival disadvantage in the LVEF < 20% subgroup was evident within the first postoperative year and persisted at 24 and 36 months, with consistently inferior survival throughout the follow-up period. The number-at-risk table illustrates the gradual reduction in patients available for follow-up at 12, 24, and 36 months (Figure 6).

### 3.3. Independent Predictors of In-Hospital Mortality

Univariate logistic regression analysis of baseline characteristics for age, BMI, sex, diabetes mellitus, arterial hypertension, lung disease (including COPD), neurological disease, or preoperative renal failure was performed (Table 9). None of these factors demonstrated a statistically significant association with in-hospital mortality (all *p* > 0.05).

In the combined regression analyses (Table 10), several variables were significantly associated with in-hospital mortality on univariate testing and remained independent predictors in the multivariate model. These included: preoperative troponin positivity (adjusted OR 2.12; *p* = 0.042), preoperative inotrope use (adjusted OR 3.88; *p* = 0.004), NYHA class IV (adjusted OR 3.72; *p* = 0.005), CCS class IV (adjusted OR 2.97; *p* = 0.011), EuroSCORE II > 8% (adjusted OR 3.89; *p* = 0.003), LVEF < 20% (adjusted OR 2.88; *p* = 0.015), and myocardial infarction within <2 days (adjusted OR 4.67; *p* = 0.001). Intra-/perioperative predictors that remained significant were emergency surgery under CPR (adjusted OR 6.13; *p* = 0.001), MCS device use (adjusted OR 5.42; *p* = 0.001), single-vessel revascularization (1 vs. ≥3 grafts) (adjusted OR 2.48; *p* = 0.038), and longer bypass time (per 10 min increase; adjusted OR 1.14; *p* = 0.021). Postoperative complications independently associated with mortality included acute renal failure (adjusted OR 2.96; *p* = 0.012), dialysis (adjusted OR 3.87; *p* = 0.003), severe bleeding (adjusted OR 2.94; *p* = 0.018), and postoperative vascular complications (adjusted OR 3.41; *p* = 0.021).

## 4. Discussion

This single-centre retrospective study evaluated early and mid-term outcomes of isolated coronary artery bypass grafting (CABG) in patients with severely reduced left ventricular ejection fraction (LVEF ≤ 30%). The main findings indicate that CABG in this high-risk population is associated with substantial perioperative morbidity and mortality; however, patients who survive the early postoperative phase demonstrate meaningful mid-term survival. These observations are consistent with randomized and observational data obtained from patients with ICM [8,13,14,23].

Compared with the STICH population (EF ≤ 35%), our patients were older (mean ~67 vs. ~60 years), more frequently underwent urgent or emergency surgery, and more often presented with recent MI or cardiogenic decompensation [13,24]. Similarly, in STICHES, long-term survival improved with CABG, but the perioperative risk profile was less severe than in our cohort [14]. Thus, our findings extend trial evidence to a substantially higher-risk real-world population treated at a tertiary referral centre, introducing both selection bias and referral bias, which limits direct comparability with randomized trials.

Large registry studies confirm that CABG patients with severe LVD commonly exhibit a high comorbidity burden. Topkara et al. reported male predominance (77%), high diabetes rates (~40%), and extensive multivessel disease [20]. Hillis et al. observed higher rates of renal dysfunction, COPD, and urgent presentation in EF < 30% cohorts [25]. Najafi et al. identified diabetes, renal dysfunction, and pulmonary disease as key predictors of adverse outcomes [18], and Jameie et al. demonstrated synergistic effects of hypertension and diabetes on survival [26]. Registry-based analyses further emphasize the impact of multimorbidity on long-term mortality [27,28], with older age, higher EuroSCORE II, and frequent heart failure admissions consistently reported [29,30].

Our baseline characteristics mirror international experience but reflect an even more acute case mix. The high proportion of urgent and emergency operations, including surgery under CPR, underscores the real-world setting and partly explains the elevated early mortality observed. Surgical urgency was striking: 40.8% of patients were urgent, 21.8% were emergency, and 14.3% were operated on under ongoing CPR. Additionally, 76.9% were NYHA III–IV, 34.0% were CCS IV, 56.5% had EuroSCORE II > 8%, 59.9% were troponin-positive, and 33.3% required inotropes rates far exceeding those in STICH, where >80% were elective and <5% emergencies [13,24]. In the New York State registry, only 9% of EF ≤ 30% patients were emergency cases [20]. These differences highlight the instability and operative complexity of our cohort.

Mechanical circulatory support (MCS) was required in 27.9% of patients, including ECMO in 19%. Hillis et al. reported IABP use in 23% but no ECMO [25], while recent European series describe ECMO rates of 10–12% in very low EF cohorts [31]. Our higher rate reflects the extreme acuity of our cohort, particularly CPR cases.

Despite technical advances [16,17], postoperative morbidity remained substantial. LCOS was the most frequent complication, often requiring MCS. Acute renal failure, pneumonia, and arrhythmias were common and strongly associated with mortality. These findings are consistent with prior reports identifying postoperative organ dysfunction, especially renal failure and LCOS, as major determinants of early death in severe LVD [20,25,32,33].

Early mortality was concentrated in the immediate postoperative period, as demonstrated by Kaplan–Meier analyses. Although our in-hospital and 30-day mortality (≈21–22%) rates exceed those in elective cohorts [20,25,29,32,34], the temporal pattern mirrors prior studies: high early hazard followed by improved outcome. Importantly, patients who survived to hospital discharge showed a markedly improved prognosis, with acceptable survival at 12, 24, and 36 months. Similar dynamics were observed in STICH and STICHES, where long-term benefits emerged despite initial risk [13,14].

The elevated mortality observed in our cohort is likely explained by the high proportion of urgent and emergency cases (62.6%), the frequent need for preoperative cardiopulmonary resuscitation (14.3%), advanced heart failure (76.9% NYHA III–IV), and high operative risk (EuroSCORE II > 8% in 56.5%), reflecting a highly selected and clinically unstable real-world population.

Multivariate analysis identified preoperative instability (positive troponin, inotrope use, CPR, advanced NYHA/CCS class, EuroSCORE II > 8%, and LVEF < 20%) as a strong predictor of in-hospital mortality. These findings align with previous series [35]. Hillis et al. demonstrated that preoperative shock and inotrope dependence predicted early death in patients with EF < 30% [25], while Topkara et al. highlighted emergent surgery and CPR as risk factors [20]. Filsoufi et al. reported NYHA IV and renal dysfunction as independent risk factors associated with adverse outcomes [32,36].

Intra-operative factors such as emergency surgery under CPR, single-graft revascularization, prolonged cardiopulmonary bypass time, and ECMO requirement further increased mortality risk. Incomplete revascularization has repeatedly been associated with worse outcomes in ischemic cardiomyopathy [37,38], consistent with findings from SYNTAX and FREEDOM emphasizing completeness of revascularization [39,40]. Lack of LITA use is linked to inferior survival [41], and prolonged bypass time correlates with renal failure and LCOS in EF ≤ 30% cohorts [32,42]. The ECMO requirement reflects refractory shock and carries mortality rates exceeding 50% in most reports [43]. Postoperative complications, particularly acute renal failure, dialysis, severe bleeding, and vascular injury, emerged as powerful mortality predictors. Renal dysfunction is among the most robust predictors after CABG in low-EF patients [32,44], and severe bleeding or re-operation is consistently associated with worse survival [45]. Subgroup analysis showed that patients with LVEF < 20% experienced significantly worse early and mid-term outcomes than those with LVEF 20–30%. They required MCS more frequently and had higher rates of LCOS, renal failure, and early death. Similar thresholds have been described previously [20,25,32,34]. Nevertheless, survivors in this subgroup achieved meaningful mid-term survival, supporting individualized decision-making rather than exclusion based solely on EF.

Clinically, these findings support guideline recommendations favouring CABG in ischemic cardiomyopathy with suitable anatomy, while emphasizing meticulous risk stratification and Heart Team decision-making.

The high complication burden underscores the importance of optimized perioperative management; furthermore, Heart Team meetings play an important role in determining the best method of treatment in this fragile population. Such an approach allows integration of clinical status, coronary anatomy, operative risk, and potential benefits when selecting between surgical and alternative treatment strategies [8,11,15].

Strengths of this study include the granular perioperative dataset, uniform inclusion of patients with LVEF ≤ 30% undergoing isolated CABG, and detailed subgroup analysis (≤20% vs. 20–30%). The cohort reflects a real-world, high-risk population underrepresented in randomized trials [13]. Use of multivariate models integrating pre-, intra-, and postoperative variables strengthens the robustness of the findings.

Limitations include the retrospective single-centre design, potential selection bias, and limited sample size for rare events. The absence of a PCI or medical therapy control group precludes direct strategy comparison.

Detailed assessments of coronary complexity (e.g., SYNTAX score), myocardial viability testing, and completeness of revascularization were not consistently available and therefore could not be analyzed, representing an additional limitation of this study.

Moreover, ethnicity was not recorded in this study. Although our centre serves a multicultural population, ethnicity-specific analyses were not possible, which may limit the generalizability of the findings.

In addition, given the limited number of in-hospital deaths, the multivariable regression analysis should be interpreted as exploratory, and the possibility of model overfitting cannot be excluded.

Therefore, our findings should not be interpreted as evidence of superiority of surgical revascularization over alternative strategies, but rather as real-world outcome data in a highly selected, high-risk population.

Contemporary management of patients with ischemic cardiomyopathy extends beyond revascularization and includes guideline-directed medical therapy (GDMT), which has been shown to improve survival and reduce sudden cardiac death. In the present study, detailed data on GDMT, arrhythmic risk stratification, and device therapy (e.g., ICD implantation) were not systematically available due to the retrospective design. Therefore, the interaction between surgical revascularization and modern heart failure therapy could not be fully assessed and should be addressed in future prospective studies integrating pharmacological and device-based treatment strategies.

## 5. Conclusions

In conclusion, CABG in patients with severely reduced left ventricular function remains associated with substantial perioperative risk, particularly in those with very low LVEF and preoperative clinical instability. However, patients who survive the early postoperative phase demonstrate relevant mid-term survival, supporting surgical revascularization as a valid treatment option in carefully selected patients with ischemic cardiomyopathy, in line with the current evidence and guideline recommendations.

These findings underscore the importance of careful patient selection, comprehensive risk stratification, and optimization of guideline-directed medical therapy, as well as the central role of multidisciplinary Heart Team decision-making.

Importantly, the absence of a comparator group (percutaneous coronary intervention or optimal medical therapy) limits the ability to draw conclusions regarding the relative benefit of surgical revascularization.

Future prospective and comparative studies incorporating contemporary medical therapy and standardized risk stratification are warranted to better define optimal management strategies in this complex patient population.

In addition, the role of contemporary heart failure therapy, including GDMT, sudden cardiac death prevention, and device-based strategies, should be considered when interpreting these findings.

These findings may support clinical decision-making by helping to identify patients who are most likely to benefit from surgical revascularization despite elevated perioperative risk.

## Figures and Tables

**Table 1:** Distribution of baseline comorbidities according to LVEF subgroups.

Variable	EF < 20% (*n* = 43, 29.3%)	EF 20–30% (*n* = 104, 70.7%)	Total (%) *n* = 147	*p*-Value
Diabetes Mellitus	19 (44.2%)	44 (42.3%)	63 (42.9%)	0.267
Hypertension	23 (53.5%)	64 (61.5%)	87 (59.2%)	0.056
Lung Disease (COPD)	8 (18.6%)	13 (12.5%)	21 (14.2%)	0.232
Neurologic Disease	4 (9.3%)	11 (10.6%)	15 (10.2%)	0.576
Pre-op. Renal failure	6 (14%)	6 (5.8%)	12 (8.2%)	0.068
Mean ± SD (Median) [Range]				
BMI Level	26.95 ± 5.4 (25.62) [18–46]	27.70 ± 4.4 (27.44) [19–40]		0.504

Data are presented as mean ± SD (median) [range], where appropriate, or as numbers (%). COPD = chronic obstructive pulmonary disease; Pre-op. = preoperative; BMI = Body Mass Index.

**Table 2:** Distribution of patients according to the number of preoperative risk factors.

Exact N Risk Factors	Exact Patients	Exact Percentage (%)	At Least N Risk Factors	Cumulative Patients	Cumulative Percentage (%)
0	9	6.1	0	147	100.0
1	36	24.5	1	138	93.9
2	58	39.5	2	102	69.4
3	33	22.4	3	44	29.9
4	10	6.8	4	11	7.5
5	1	0.7	5	1	0.7

The table shows both the exact and cumulative distribution of patients according to the number of preoperative risk factors (diabetes mellitus, arterial hypertension, lung disease, neurological disease, renal disease, and Body Mass Index (BMI) ≥ 25).

**Table 3:** Preoperative clinical characteristics of patients according to LVEF subgroup.

Variable	EF < 20% *n* = 43 (29.3%)	EF 20–30% *n* = 104 (70.7%)	Total (%) *n* = 147	*p*-Value
Troponin (Pre-op.)	32 (74.4%)	56 (53.8%)	88 (59.9%)	0.025
Use of Inotropic agents (Pre-op.)	23 (53.5%)	26 (25.0%)	49 (33.3%)	0.001
NYHA Classification				0.001
I	1 (2.3%)	4 (3.8%)	5 (3.4%)	
II	6 (14.0%)	23 (22.1%)	29 (19.7%)	
III	12 (27.9%)	44 (42.3%)	56 (38.1%)	
IV	24 (55.8%)	33 (31.7%)	57 (38.8%)	
CCS Classification				0.002
0	2 (4.7%)	19 (18.3%)	21 (14.3%)	
I	2 (4.7%)	11 (10.6%)	13 (8.8%)	
II	8 (18.6%)	15 (14.4%)	23 (15.6%)	
III	7 (16.3%)	33 (31.7%)	40 (27.2%)	
IV	24 (55.8%)	26 (25.0%)	50 (34.0%)	
Euroscore II Group				0.001
0–4% (low risk)	5 (11.6%)	36 (34.6%)	41 (27.9%)	
4–8% (middle risk)	9 (20.9%)	14 (13.5%)	23 (15.6%)	
>8% (high risk)	29 (67.4%)	54 (51.9%)	83 (56.5%)	

Data are presented as numbers (%). Pre-op. = preoperative; NYHA = New York Heart Association functional class; CCS = Canadian Cardiovascular Society.

**Table 4:** Distribution of patients’ cardiac characteristics according to LVEF subgroup.

Variable	EF < 20% *n* = 43 (29.3%)	EF 20–30% *n* = 104 (70.7%)	Total (%) *n* = 147	*p*-Value
Myocardial infarction				0.007
No	8 (18.6%)	32 (30.8%)	40 (27.2%)	
Yes (<2 days)	25 (58.1%)	37 (35.6%)	62 (42.2%)	
Yes (2–21 days)	5 (11.6%)	16 (15.4%)	21 (14.3%)	
Cardiac arrhythmia (Pre-op.)	6 (14.0%)	19 (18.4%)	25 (17.0%)	0.695
Left main stem disease (LMS; ≥50% LMS stenosis)	24 (55.8%)	52 (50.0%)	76 (51.7%)	0.229
Previous PCI	11 (25.6%)	22 (21.2%)	33 (22.4%)	0.323
Carotid artery stenosis	5 (11.6%)	18 (17.3%)	23 (15.6%)	0.934
Coronary artery disease				0.931
1 Vessel	1 (2.3%)	2 (1.9%)	3 (2.0%)	
2 Vessel	1 (2.3%)	5 (4.8%)	6 (4.1%)	
3 Vessel	41 (95.3%)	97 (93.3%)	138 (93.9%)	

Data are presented as numbers (%). Pre-op. = preoperative; LMS = left main stem; PCI = percutaneous coronary intervention.

**Table 5:** Association between preoperative LVEF and intra-operative surgical information.

Variable	Pre LVEF (%)			Total (%) *n* = 147	*p*-Value
	EF < 20% *n* = 43	EF 20–30% *n* = 104			
Type of Surgery					0.001
Elective	3 (7.0)	31 (29.8)		34 (23.1)	
Urgent	15 (34.9)	45 (43.3)		60 (40.8)	
Emergency	10 (23.3)	22 (21.2)		32 (21.8)	
Emergency under reanimation	15 (34.9)	6 (5.8)		21 (14.3)	
Number of grafts used					0.251
1	4 (9.3)	6 (5.8)		10 (6.8)	
2	18 (41.9)	32 (30.8)		50 (34)	
3 or 4	21 (48.8)	66 (63.4)		87 (59.2)	
Internal thoracic artery graft	39 (90.7)	97 (93.3)		136 (92.5)	0.59
Heart–lung machine (HLM)	35 (81.4)	82 (78.8)		117 (79.6)	0.727
Mechanical circulatory support	22 (51.2)	19 (18.3)		41 (27.9)	0.007
Mean ± SD (Range)					
Duration of support device (days)	2.33 ± 3.2		1.14 ± 3.4		0.046
Duration of aortic clamp (min.)	36.49 ± 38.9 (23–111)		39.31 ± 34.5 (16–105)		0.681
Duration of bypass (min.)	119.67 ± 72.0 (47–330)		87.7 ± 53.4 (28–200)		0.011

Data are presented as mean ± SD (range), where appropriate, or as numbers (%). min. = minutes.

**Table 6:** Hospitalization information according to LVEF subgroup.

Variable	EF < 20% *n* = 43 (29.3%)	EF 20–30% *n* = 104 (70.7%)	*p*-Value
Mean ± SD (Median) [Range]			
Intensive care unit (d)	8.1 ± 7.6 (6) [1–31]	6.4 ± 7.7 (4) [1–38]	0.033
Hospital LOS (d)	12.2 ± 8.5 (9) [1–36]	15.4 ± 11.3 (12) [2–86]	0.029

Data are presented as mean ± SD (median) [range]. LOS = length of stay; d = days.

**Table 7:** Postoperative complications according to LVEF subgroup.

Complication	EF < 20% *n* = 43 (29.3%)	EF 20–30% *n* = 104 (70.7%)	All Patients (*n* = 147)	*p*-Value
Low Cardiac Output Syndrome (Post-Op.)	36 (83.7%)	57 (54.8%)	93 (63.3%)	0.001
Acute Renal Failure (Post-Op.)	18 (41.9%)	24 (23.1%)	42 (28.6%)	0.028
Renal Replacement Therapy (Post-Op.)	16 (37.2%)	21 (20.2%)	37 (25.2%)	0.038
Pneumonia (Post-Op.)	17 (39.5%)	22 (21.2%)	39 (26.5%)	0.026
Complications requiring Emergency Re-Operation	15 (34.9%)	14 (13.5%)	29 (19.7%)	0.005
Arrhythmia (New-Onset)	11 (25.6%)	23 (22.1%)	34 (23.1%)	0.671
Life-Threatening Post-Op. Bleeding	11 (25.6%)	8 (7.7%)	19 (12.9%)	0.006
Cerebral/Cerebrovascular Event (Any)	6 (13.9%)	11 (10.6%)	17 (11.6%)	0.902
Sternum Instability	3 (7.0%)	4 (3.8%)	7 (4.8%)	0.417
Wound Healing Problem (Any Site)	3 (7.0%)	8 (7.7%)	11 (7.5%)	0.637
Bypass Revision	3 (7.0%)	1 (1.0%)	4 (2.7%)	0.075
Mediastinitis (KISS)	1 (2.3%)	1 (1.0%)	2 (1.4%)	0.501
Post-Op. Ischemia (Intestinal or Leg)	0 (0%)	6 (5.8%)	6 (4.1%)	0.274

Data are presented as numbers (%). Post-op. = postoperative; KISS = Hospital Infection Surveillance System (Krankenhaus-Infektions-Surveillance-System).

**Table 8:** Mortality outcomes according to LVEF subgroup.

Outcome/Follow-Up	All Patients (*n* = 147)	EF < 20% (*n* = 43)	EF 20–30% (*n* = 104)	*p*-Value
In-hospital mortality	31 (21.1%)	16 (37.2%)	15 (14.4%)	0.002
30-day mortality	35 (23.8%)	18 (41.9%)	17 (16.3%)	<0.001
12-month mortality	46 (31.3%)	22 (51.2%)	24 (23.1%)	0.005
24-month mortality	55 (37.4%)	24 (55.8%)	31 (29.8%)	
36-month mortality	63 (42.9%)	25 (58.1%)	38 (36.5%)	

Data are presented as numbers (%). *p*-values refer to between-group comparisons for mortality.

**Table 9:** Univariate analysis of preoperative baseline risk factors for in-hospital mortality.

Variable	Univariate OR (95% CI)	*p*-Value
Age	1.01 (0.975–1.05)	0.529
BMI	0.974 (0.901–1.05)	0.514
Gender (M/F)	0.564 (0.253–1.26)	0.156
Diabetes mellitus	1.07 (0.821–1.39)	0.628
Arterial hypertension	1.69 (0.8–3.57)	0.169
Lung disease (incl. COPD)	1.27 (0.843–1.91)	0.254
Neurological disease	1.25 (0.437–3.56)	0.679
Preoperative renal failure	1.16 (0.635–2.12)	0.627

Univariate logistic regression was performed for each baseline preoperative variable. Values are presented as odds ratios with 95% confidence intervals. A two-sided *p* < 0.05 was considered statistically significant. OR = odds ratio; CI = confidence interval; BMI = Body Mass Index.

**Table 10:** Univariate and multivariate logistic regression analysis of independent predictors of in-hospital mortality.

Variable	Univariate OR (95% CI)	*p*-Value	Multivariate OR (95% CI)	*p*-Value
**Preoperative troponin positive**	2.65 (1.12–6.25)	0.025	2.12 (1.01–4.92)	0.042
**Preoperative inotrope use**	5.36 (2.31–12.4)	0.001	3.88 (1.54–9.22)	0.004
**NYHA class IV**	4.58 (1.98–10.1)	0.001	3.72 (1.48–9.12)	0.005
**CCS class IV**	3.91 (1.67–8.65)	0.002	2.97 (1.29–7.13)	0.011
**EuroSCORE > 8%**	5.24 (2.14–12.2)	0.001	3.89 (1.56–9.63)	0.003
**Preoperative EF < 20%**	3.49 (1.56–7.89)	0.002	2.88 (1.20–6.92)	0.015
**Myocardial infarction (<2 days)**	6.11 (2.35–13.7)	0.001	4.67 (1.88–11.2)	0.001
**Emergency Op. under reanimation**	8.25 (3.14–18.9)	0.001	6.13 (2.45–14.7)	0.001
**Cardiac support device use**	7.29 (2.81–16.5)	0.001	5.42 (2.01–12.7)	0.001
**Number of grafts (1 vs. ≥3)**	3.12 (1.20–8.11)	0.025	2.48 (1.05–6.77)	0.038
**Bypass time (per 10 min. increase)**	1.19 (1.04–1.35)	0.014	1.14 (1.02–1.28)	0.021
**Acute renal failure (Post-op.)**	3.89 (1.77–8.67)	0.001	2.96 (1.28–7.11)	0.012
**Dialysis (Post-op.)**	5.14 (2.24–11.6)	0.001	3.87 (1.56–9.38)	0.003
**Severe bleeding (Post-op.)**	4.12 (1.63–9.78)	0.003	2.94 (1.19–7.25)	0.018
**Post-op. vascular complication**	4.93 (1.29–11.8)	0.009	3.41 (1.12–8.77)	0.021

Univariate logistic regression was performed for each preoperative, intra-operative, and postoperative variable. Variables with *p* < 0.20 in univariate analysis, or with established clinical relevance, were entered into the multivariate logistic regression model. The number of grafts was coded as 1 vs. ≥3 grafts, and bypass times were analyzed as continuous variables. A two-sided *p* < 0.05 was considered statistically significant. OR = odds ratio; CI = confidence interval; NYHA = New York Heart Association Classification; CCS = Canadian Cardiovascular Society Classification; EF = ejection fraction; min. = minutes; post-op. = postoperative.

## Data Availability

The original contributions presented in this study are included in the article. Further inquiries can be directed to the corresponding author.

## Abbreviations

The following abbreviations are used in this manuscript:

ACC	American College of Cardiology
AHA	American Heart Association
BMI	Body Mass Index
CABG	Coronary Artery Bypass Grafting
CAD	Coronary Artery Disease
CCS	Canadian Cardiovascular Society
CI	Confidence Interval
COPD	Chronic Obstructive Pulmonary Disease
CPR	Cardiopulmonary Resuscitation
ECMO	Extracorporeal Membrane Oxygenation
EACTS	European Association for Cardio-Thoracic Surgery
EF	Ejection Fraction
ESC	European Society of Cardiology
EuroSCORE II	European System for Cardiac Operative Risk Evaluation II
GDMT	Guideline-Directed Medical Therapy
IABP	Intra-Aortic Balloon Pump
ICM	Ischemic Cardiomyopathy
ICU	Intensive Care Unit
KISS	Krankenhaus-Infektions-Surveillance-System
LCOS	Low Cardiac Output Syndrome
LITA	Left Internal Thoracic Artery
LMS	Left Main Stem
LOS	Length of Stay
LV	Left Ventricle
LVAD	Left Ventricular Assist Device
LVD	Left Ventricular Dysfunction
LVEF	Left Ventricular Ejection Fraction
MCS	Mechanical Circulatory Support
MI	Myocardial Infarction
NYHA	New York Heart Association
OMT	Optimal Medical Therapy
OR	Odds Ratio
PCI	Percutaneous Coronary Intervention
RVAD	Right Ventricular Assist Device
SD	Standard Deviation
TTE	Transthoracic Echocardiography
